# Papillary Thyroid Carcinoma With a Rare TFG-MET Fusion in a Chernobyl Survivor: A Case Report

**DOI:** 10.7759/cureus.85602

**Published:** 2025-06-09

**Authors:** Abeer Jacob, Hiren J Patel, Sameer Andani, Xiaoyin Sara Jiang

**Affiliations:** 1 Pathology, Trinity Medical Sciences University School of Medicine, Roswell, USA; 2 Internal Medicine, Nova Southeastern University Dr. Kiran C. Patel College of Osteopathic Medicine, Fort Lauderdale, USA; 3 Pathology, University of South Florida, Tampa, USA; 4 Head and Neck Pathology, Tampa General Hospital, Tampa, USA

**Keywords:** chernobyl, follicular variant of papillary thyroid carcinoma, head and neck cancer pathology, ionising radiation exposure, papillary cancer of thyroid, thyroid cancer

## Abstract

Papillary thyroid carcinoma (PTC) is the most common malignant thyroid tumor, with several histologic subtypes, including the follicular subtype (follicular variant of papillary thyroid carcinoma (FVPTC)). While *BRAF V600E* mutations are frequently associated with PTC, alternative molecular alterations have been identified, particularly following radiation. Here, we present a case of FVPTC in a 43-year-old female with a history of childhood nuclear radiation exposure from the Chernobyl disaster. Although radiation exposure is a known risk factor for thyroid malignancies, the molecular features of such cases remain an area of ongoing study. Sequencing of this patient's tumor revealed a *TFG-MET *fusion, a rare genetic alteration that has been reported in only isolated cases of thyroid carcinoma. *MET* fusions are not well characterized in thyroid tumors, especially in the context of radiation exposure. This case contributes to the limited literature on kinase fusion-driven thyroid carcinomas, particularly those with follicular architecture arising after radiation exposure. Recognizing such rare genetic findings may help refine our understanding of the molecular diversity seen in radiation-associated thyroid cancers.

## Introduction

Papillary thyroid carcinoma (PTC) is the most common malignant thyroid tumor, accounting for approximately 80% of all thyroid cancers [1,2]. Radiation exposure is a well-established risk factor for thyroid cancer, with increased incidence of PTC in populations exposed to nuclear fallout, such as survivors of the Chernobyl disaster [1].Ionizing radiation is thought to drive thyroid carcinogenesis primarily through the induction of double-strand DNA breaks, which can lead to chromosomal rearrangements and the formation of oncogenic gene fusions [3]. Among these, *RET/PTC* and *NTRK *rearrangements are well-documented in radiation-associated thyroid cancers and are considered key molecular drivers in this context.

While such fusions are relatively common in radiation-induced thyroid cancers, the *TGF-MET* fusion represents an exceptionally rare molecular alteration. Though previously described in other solid tumors, including lung cancer, it is exceedingly uncommon in thyroid malignancies [4-13]. The *MET* proto-oncogene, which promotes cell proliferation and survival, can become constitutively active when fused with *TFG *[14].

Here, we report a case of follicular subtype of PTC (follicular variant of papillary thyroid carcinoma (FVPTC)) in a 43-year-old female with a history of childhood nuclear radiation exposure from the Chernobyl accident. Molecular profiling of the tumor revealed a rare *TFG-MET* fusion. While FVPTC is a common histological subtype, radiation-associated and kinase fusion-driven cases remain uncommon [6]. This report adds to the understanding of the molecular alterations that can arise in this unique setting, particularly in fusion-driven tumors with follicular architecture.

## Case presentation

A 43-year-old woman presented to a tertiary care hospital with a progressively enlarging left thyroid mass over one year, associated with compressive symptoms including dysphagia. The patient had a childhood history of exposure to nuclear radiation during the Chernobyl nuclear incident. A fine needle aspiration (FNA) biopsy of the left thyroid nodule was performed, demonstrating cytology consistent with a Bethesda category IV nodule, suspicious for a follicular neoplastic process [15]. Following this, the patient underwent a total thyroidectomy with bilateral paratracheal lymph node dissection.

**Figure 1:**
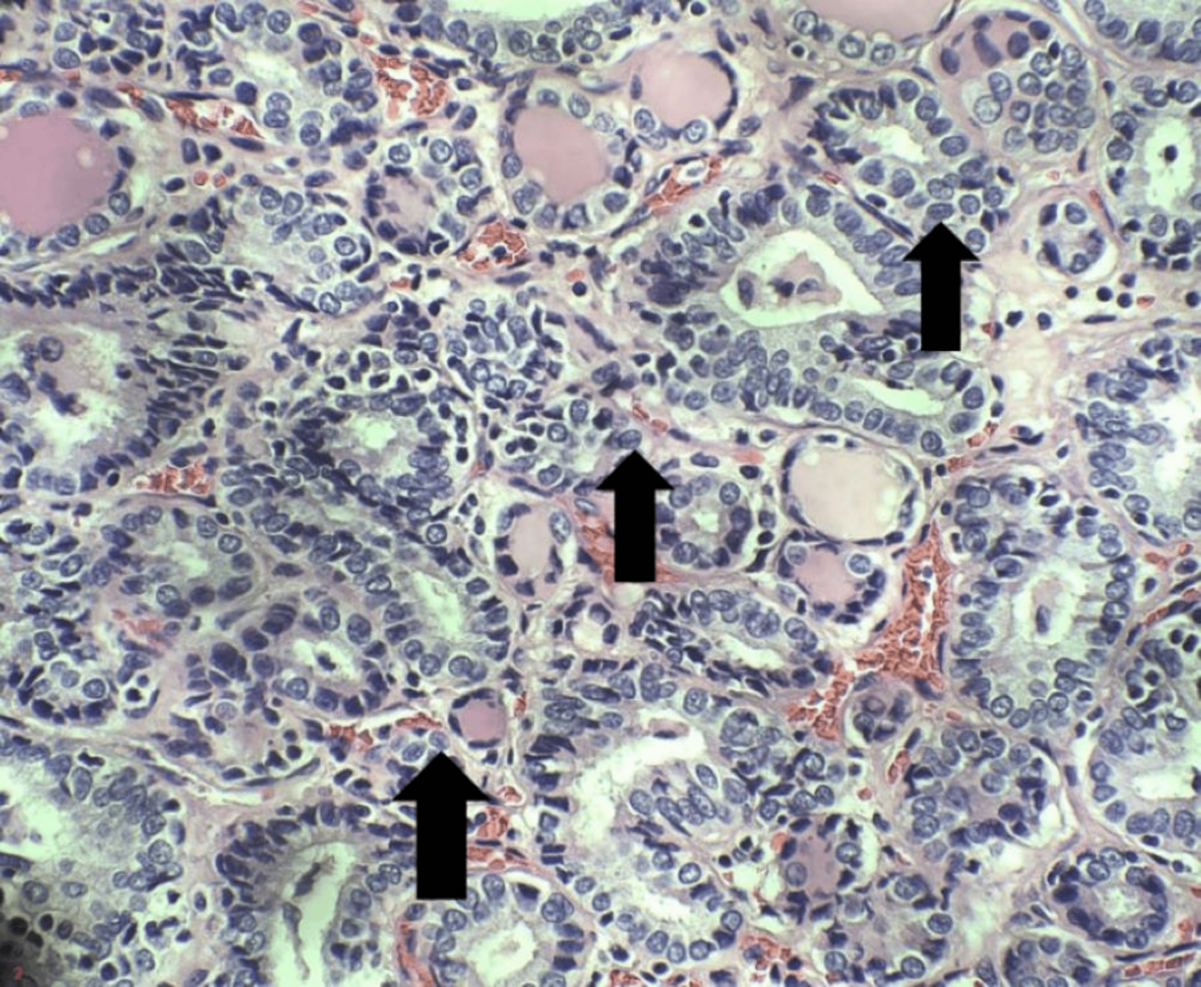
Immunohistochemical staining High-power view reveals nuclear features typical of infiltrative FVPTC, including crowded overlapping nuclei, nuclear grooves (arrows), pseudoinclusions, and vacuoles (H&E, 40x).

Gross examination of the thyroidectomy specimen revealed two lesional foci: a 3.2 cm nodule in the left lobe and a 0.2 cm nodule on the right lobe. Sectioning of the left lobe revealed a dominant, unencapsulated nodule located in the inferior pole that was tan-pink and soft and showed a focal peripheral yellow area of firm discoloration. The right lobe exhibited a more uniformly mottled cut surface with a small central yellow area. Microscopically, both nodules demonstrated a follicular architectural pattern accompanied by pathognomonic features of PTC, including a constellation of nuclear characteristics such as overlapping oval to round nuclei, nuclear grooves, pseudoinclusions, and clearing and clumping of chromatin (Figure 1). Scattered areas with squamous morules were seen.

**Figure 2:**
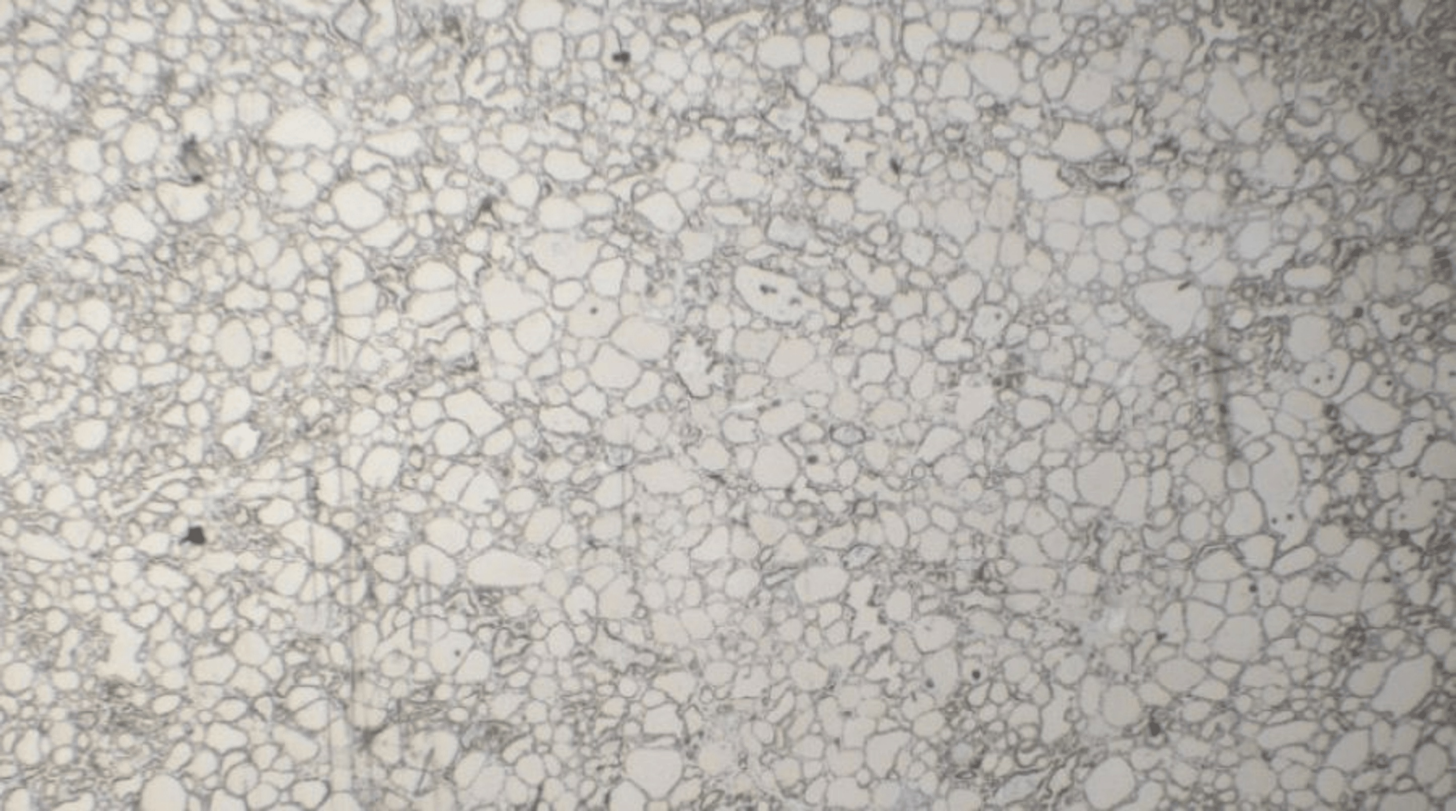
Immunohistochemical staining Low-power view shows TTF-1 positivity within the lesional cells, confirming thyroid origin (2x). While a higher-power image is not available, nuclear staining was consistently observed throughout the tumor on visual inspection.

Immunohistochemical (IHC) analysis demonstrated TTF-1 positivity (Figure 2) within the lesional cells, confirming thyroid origin, and p40 expression within squamous morules, supporting squamous differentiation in the context of a rare *TGF-MET* fusion mutation [16,17]. Vascular invasion was highlighted using CD31 and pan-cytokeratin staining [18]. No perineural invasion was identified, and all the surgical margins were negative. Bilateral paratracheal lymph nodes were both negative for metastatic carcinoma. Given the patient’s clinical history and histologic findings, molecular testing was pursued. ThyroSeq v3, a targeted next-generation sequencing (NGS) panel evaluating point mutations, gene fusions, and copy number alterations common to thyroid cancer, identified a *TFG-MET* gene fusion within the tumor sample.

## Discussion

Childhood exposure to radiation is a well-established risk factor for thyroid malignancies, including PTC [19]. This case represents a rare instance of infiltrative FVPTC likely triggered by childhood exposure to nuclear fallout from the Chernobyl disaster in April 1986. Consistent with existing literature, thyroid neoplasms that develop within the first decade after exposure are more likely to present as the solid or trabecular subtypes of PTC, whereas tumors arising after a longer latency, such as in the second decade or beyond, more frequently present as FVPTC or classic PTC [20]. This patient’s diagnosis of FVPTC several decades after childhood exposure aligns with these observations.

This case harbored a *TFG-MET *fusion, a relatively rare kinase fusion that has been identified in only a narrow range of malignancies [10]. In thyroid cancer specifically, *MET *fusions have been rarely reported. The discovery of *TFG-MET* fusion in this setting broadens the known molecular spectrum of radiation-associated thyroid carcinomas and raises important questions about the potential for underrecognized rare gene fusions to drive tumorigenesis following radiation exposure.

Although *MET *fusions are well-documented oncogenic drivers in malignancies such as renal cell carcinoma, non-small cell lung cancer, colorectal cancer, and breast cancer, their role in thyroid carcinogenesis remains poorly characterized. In these cancers, *MET *activation contributes to tumor growth, invasion, and metastasis through its effects on cell proliferation and survival [13]. Kinase fusion-related thyroid carcinomas such as this case have been broadly described as exhibiting follicular architecture, squamous morules, fibrous bands, and lymphovascular invasion, often with a clinically aggressive course involving metastases to lymph nodes and distant sites [8]. This case shows histologic features consistent with those seen in other cases with *MET *fusions [9,21,22].

Thyroid cancers following the Chernobyl disaster exhibited a notable rise in incidence, with over 6,000 cases of thyroid carcinoma reported among individuals exposed as children or adolescents by the year 2005 [23]. More recent epidemiological data from the United Nations Scientific Committee on the Effects of Atomic Radiation (UNSCEAR) indicate that approximately 20,000 cases of thyroid cancer were diagnosed between 1991 and 2015 in individuals who were under 18 years old at the time of the accident and resided in affected areas [24]. Such epidemiologic trends highlight the importance of continued surveillance and molecular characterization of thyroid tumors in radiation-exposed populations to better define the spectrum of genetic alterations driving these malignancies.

## Conclusions

This case highlights an instance of thyroid carcinoma in a patient with a history of childhood radiation exposure from the Chernobyl incident, distinguished by the presence of a *TFG-MET *fusion. While *BRAF *and *RAS *mutations are more common in thyroid cancer, the identification of this rare *MET *fusion contributes to the limited but growing body of literature on kinase fusion-driven thyroid carcinomas. *MET *fusions are well-established oncogenic drivers in other malignancies, and their presence in thyroid cancer represents an alternative mechanism to tumorigenesis. By documenting this case, we aim to expand the molecular characterization of fusion-driven thyroid tumors and highlight the importance of continued surveillance and reporting of such rare alterations.
